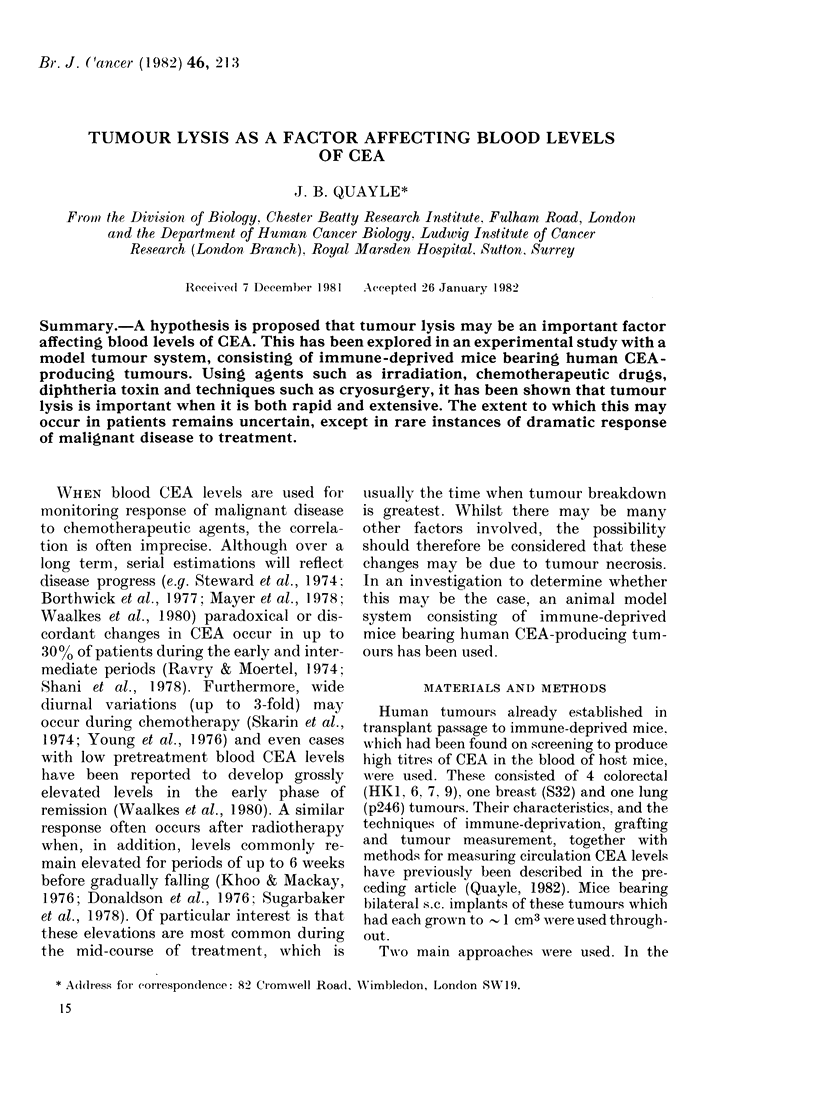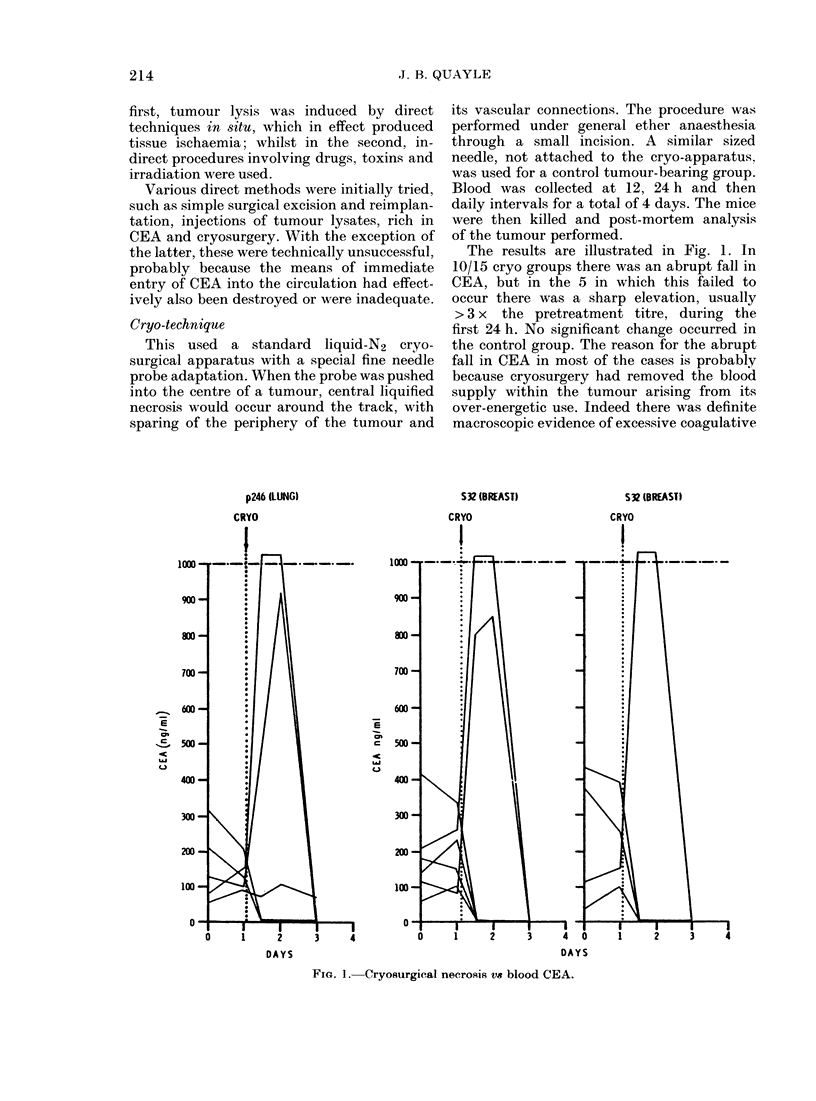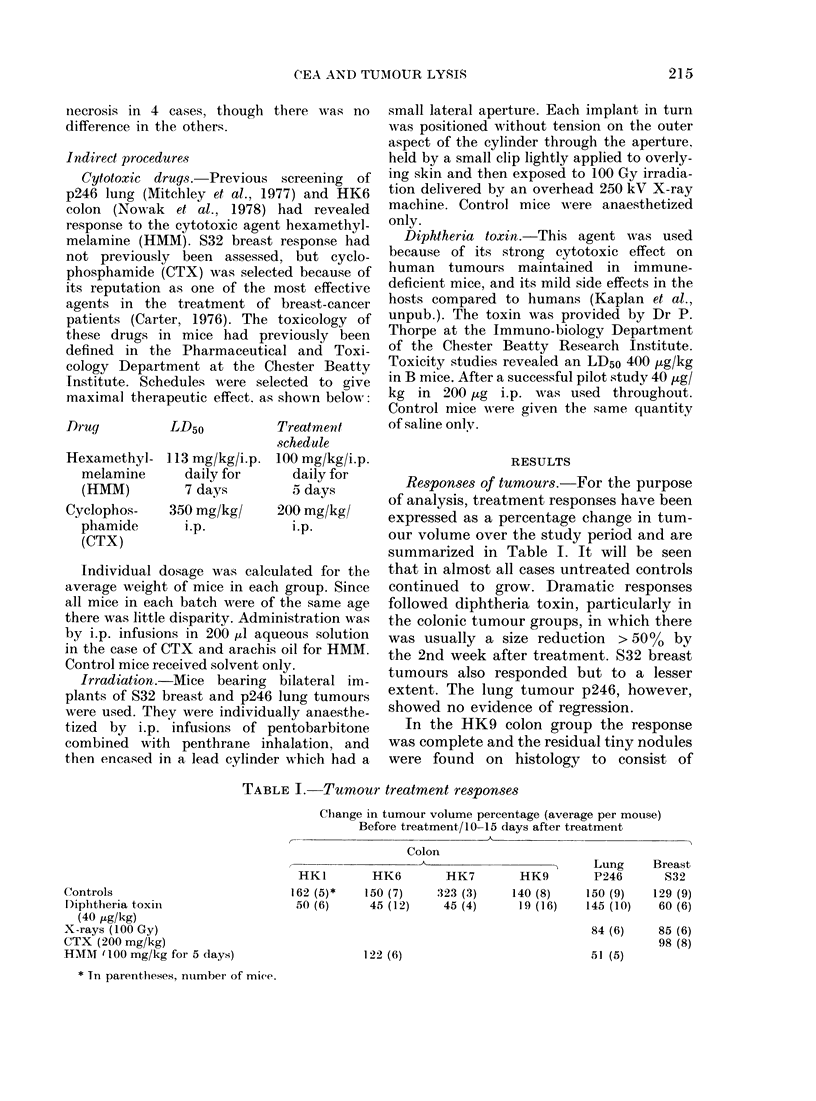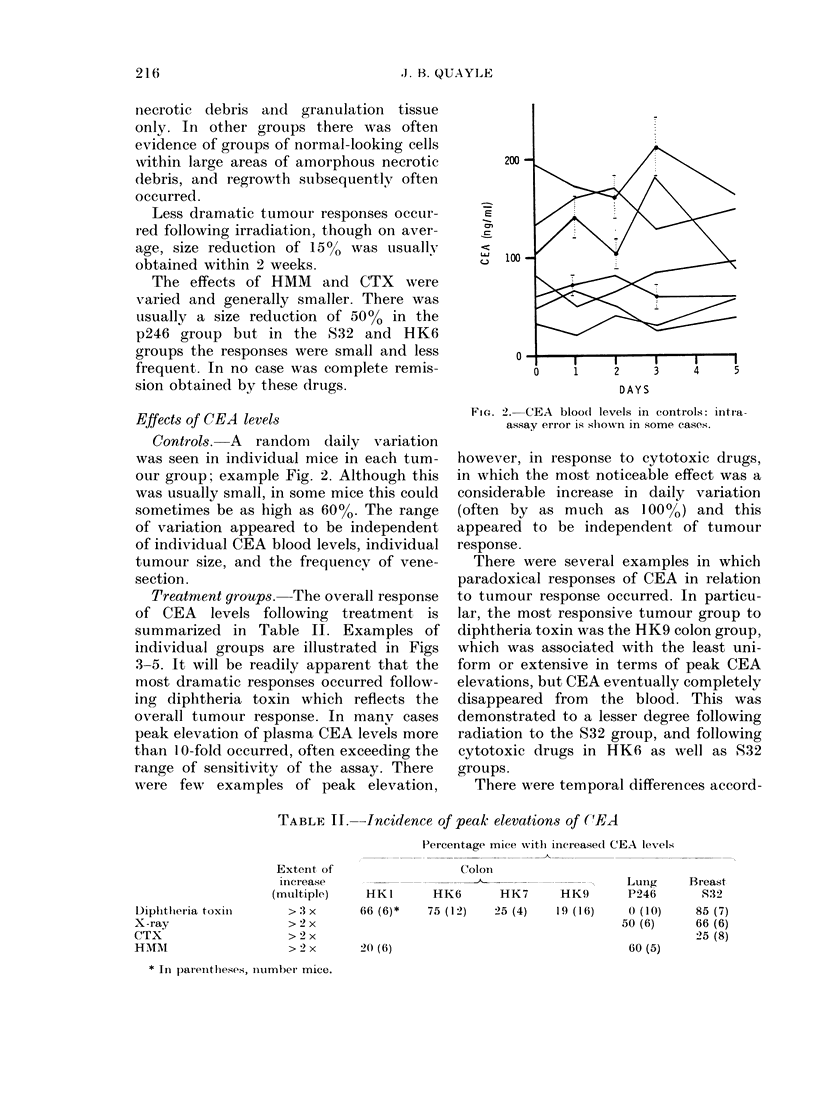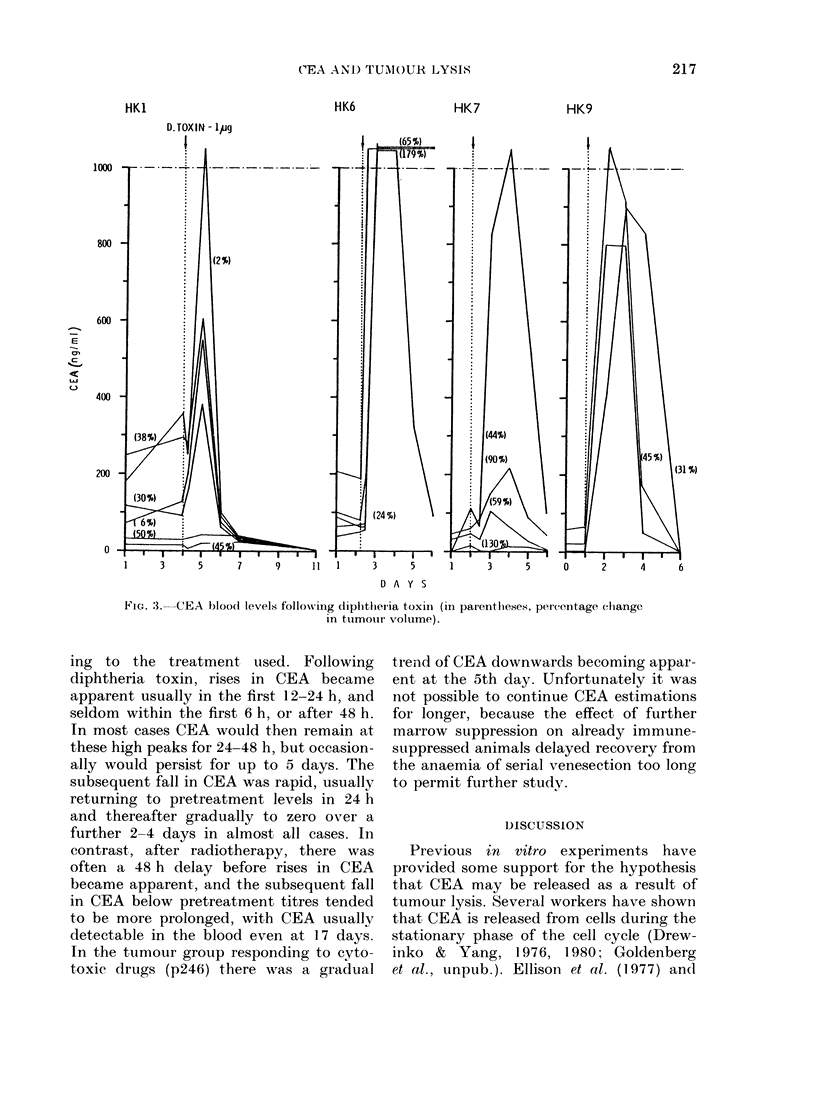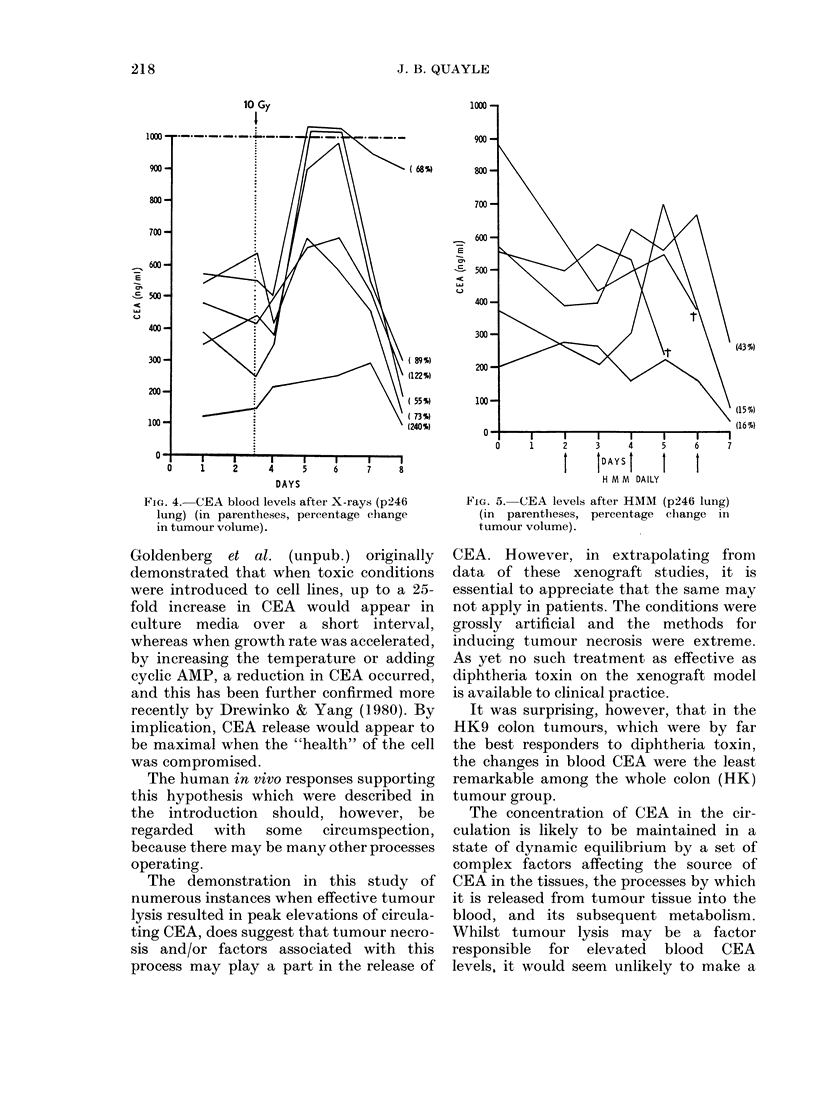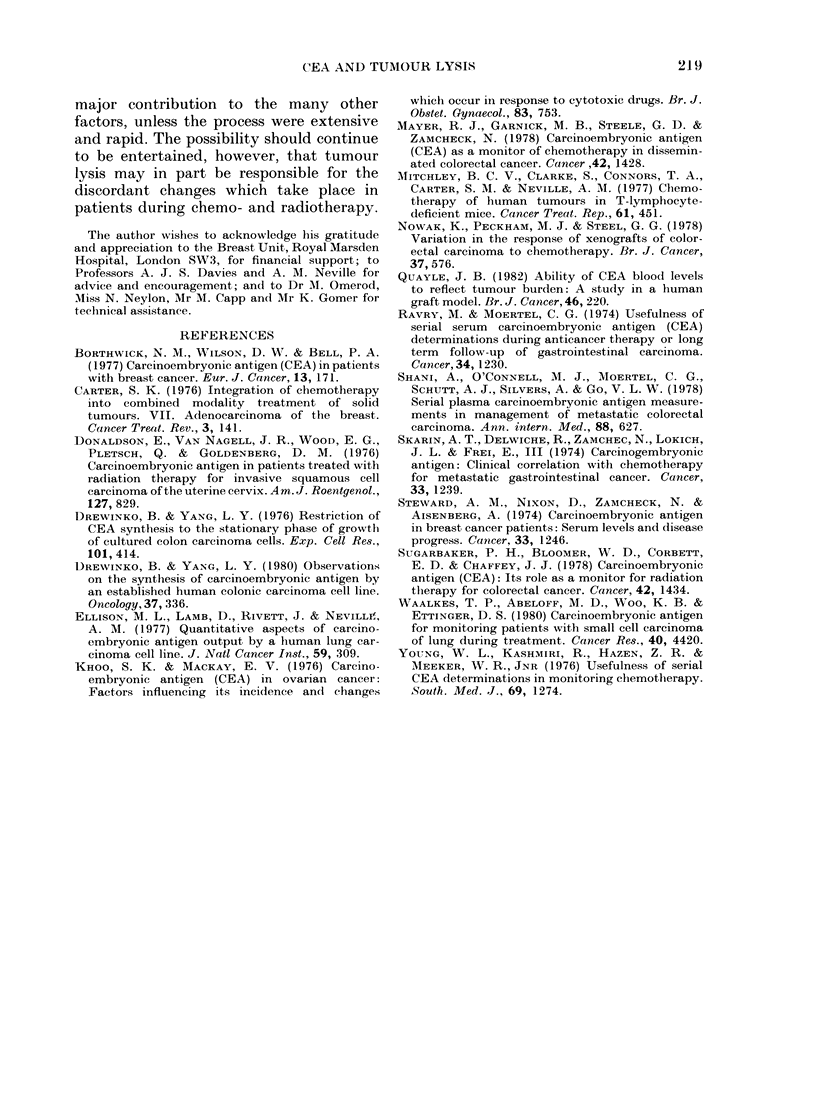# Tumour lysis as a factor affecting blood levels of CEA.

**DOI:** 10.1038/bjc.1982.186

**Published:** 1982-08

**Authors:** J. B. Quayle

## Abstract

A hypothesis is proposed that tumour lysis may be an important factor affecting blood levels of CEA. This has been explored in an experimental study with a model tumour system, consisting of immune-deprived mice bearing human CEA-producing tumours. Using agents such as irradiation, chemotherapeutic drugs, diphtheria toxin and techniques such as cryosurgery, it has been shown that tumour lysis is important when it is both rapid and extensive. The extent to which this may occur in patients remains uncertain, except in rare instances of dramatic response of malignant disease to treatment.


Br. J. Cancer (1982) 46, 213

### TUMOUR LYSIS AS A FACTOR AFFECTING BLOOD LEVELS OF CEA

J. B. QUAYLE*

*From the Division of Biology, Chester Beatty Research Institute, Fulham Road, London and the Department of Human Cancer Biology, Ludwig Institute of Cancer Research (London Branch), Royal Marsden Hospital, Sutton, Surrey*

Received 7 December 1981  Accepted 26 January 1982

**Summary.—A hypothesis is proposed that tumour lysis may be an important factor affecting blood levels of CEA. This has been explored in an experimental study with a model tumour system, consisting of immune-deprived mice bearing human CEA-producing tumours. Using agents such as irradiation, chemotherapeutic drugs, diphtheria toxin and techniques such as cryosurgery, it has been shown that tumour lysis is important when it is both rapid and extensive. The extent to which this may occur in patients remains uncertain, except in rare instances of dramatic response of malignant disease to treatment.**

When blood CEA levels are used for monitoring response of malignant disease to chemotherapeutic agents, the correlation is often imprecise. Although over a long term, serial estimations will reflect disease progress (*e.g.* Steward *et al.*, 1974; Borthwick *et al.*, 1977; Mayer *et al.*, 1978; Waalkes *et al.*, 1980) paradoxical or discordant changes in CEA occur in up to 30% of patients during the early and intermediate periods (Ravry & Moertel, 1974; Shani *et al.*, 1978). Furthermore, wide diurnal variations (up to 3-fold) may occur during chemotherapy (Skarin *et al.*, 1974; Young *et al.*, 1976) and even cases with low pretreatment blood CEA levels have been reported to develop grossly elevated levels in the early phase of remission (Waalkes *et al.*, 1980). A similar response often occurs after radiotherapy when, in addition, levels commonly remain elevated for periods of up to 6 weeks before gradually falling (Khoo & Mackay, 1976; Donaldson *et al.*, 1976; Sugarbaker *et al.*, 1978). Of particular interest is that these elevations are most common during the mid-course of treatment, which is usually the time when tumour breakdown is greatest. Whilst there may be many other factors involved, the possibility should therefore be considered that these changes may be due to tumour necrosis. In an investigation to determine whether this may be the case, an animal model system consisting of immune-deprived mice bearing human CEA-producing tumours has been used.

### MATERIALS AND METHODS

Human tumours already established in transplant passage to immune-deprived mice, which had been found on screening to produce high titres of CEA in the blood of host mice, were used. These consisted of 4 colorectal (HK1, 6, 7, 9), one breast (S32) and one lung (p246) tumours. Their characteristics, and the techniques of immune-deprivation, grafting and tumour measurement, together with methods for measuring circulation CEA levels have previously been described in the preceding article (Quayle, 1982). Mice bearing bilateral s.c. implants of these tumours which had each grown to $\sim 1$ cm$^3$ were used throughout.

Two main approaches were used. In the

* Address for correspondence: 82 Cromwell Road, Wimbledon, London SW19.

first, tumour lysis was induced by direct techniques *in situ*, which in effect produced tissue ischaemia; whilst in the second, indirect procedures involving drugs, toxins and irradiation were used.

Various direct methods were initially tried, such as simple surgical excision and reimplantation, injections of tumour lysates, rich in CEA and cryosurgery. With the exception of the latter, these were technically unsuccessful, probably because the means of immediate entry of CEA into the circulation had effectively also been destroyed or were inadequate.

*Cryo-technique*

This used a standard liquid-$N_2$ cryosurgical apparatus with a special fine needle probe adaptation. When the probe was pushed into the centre of a tumour, central liquified necrosis would occur around the track, with sparing of the periphery of the tumour and its vascular connections. The procedure was performed under general ether anaesthesia through a small incision. A similar sized needle, not attached to the cryo-apparatus, was used for a control tumour-bearing group. Blood was collected at 12, 24 h and then daily intervals for a total of 4 days. The mice were then killed and post-mortem analysis of the tumour performed.

The results are illustrated in Fig. 1. In 10/15 cryo groups there was an abrupt fall in CEA, but in the 5 in which this failed to occur there was a sharp elevation, usually $>3\times$ the pretreatment titre, during the first 24 h. No significant change occurred in the control group. The reason for the abrupt fall in CEA in most of the cases is probably because cryosurgery had removed the blood supply within the tumour arising from its over-energetic use. Indeed there was definite macroscopic evidence of excessive coagulative

FIG. 1.—Cryosurgical necrosis *vs* blood CEA.

necrosis in 4 cases, though there was no difference in the others.

*Indirect procedures*

*Cytotoxic drugs*.—Previous screening of p246 lung (Mitchley *et al*., 1977) and HK6 colon (Nowak *et al*., 1978) had revealed response to the cytotoxic agent hexamethylmelamine (HMM). S32 breast response had not previously been assessed, but cyclophosphamide (CTX) was selected because of its reputation as one of the most effective agents in the treatment of breast-cancer patients (Carter, 1976). The toxicology of these drugs in mice had previously been defined in the Pharmaceutical and Toxicology Department at the Chester Beatty Institute. Schedules were selected to give maximal therapeutic effect, as shown below:

| *Drug* | $LD_{50}$ | *Treatment schedule* |
|---|---|---|
| Hexamethylmelamine (HMM) | 113 mg/kg/i.p. daily for 7 days | 100 mg/kg/i.p. daily for 5 days |
| Cyclophosphamide (CTX) | 350 mg/kg/i.p. | 200 mg/kg/i.p. |

Individual dosage was calculated for the average weight of mice in each group. Since all mice in each batch were of the same age there was little disparity. Administration was by i.p. infusions in 200 $\mu$l aqueous solution in the case of CTX and arachis oil for HMM. Control mice received solvent only.

*Irradiation*.—Mice bearing bilateral implants of S32 breast and p246 lung tumours were used. They were individually anaesthetized by i.p. infusions of pentobarbitone combined with penthrane inhalation, and then encased in a lead cylinder which had a small lateral aperture. Each implant in turn was positioned without tension on the outer aspect of the cylinder through the aperture, held by a small clip lightly applied to overlying skin and then exposed to 100 Gy irradiation delivered by an overhead 250 kV X-ray machine. Control mice were anaesthetized only.

*Diphtheria toxin*.—This agent was used because of its strong cytotoxic effect on human tumours maintained in immune-deficient mice, and its mild side effects in the hosts compared to humans (Kaplan *et al*., unpub.). The toxin was provided by Dr P. Thorpe at the Immuno-biology Department of the Chester Beatty Research Institute. Toxicity studies revealed an $LD_{50}$ 400 $\mu$g/kg in B mice. After a successful pilot study 40 $\mu$g/kg in 200 $\mu$g i.p. was used throughout. Control mice were given the same quantity of saline only.

### RESULTS

*Responses of tumours*.—For the purpose of analysis, treatment responses have been expressed as a percentage change in tumour volume over the study period and are summarized in Table I. It will be seen that in almost all cases untreated controls continued to grow. Dramatic responses followed diphtheria toxin, particularly in the colonic tumour groups, in which there was usually a size reduction >50% by the 2nd week after treatment. S32 breast tumours also responded but to a lesser extent. The lung tumour p246, however, showed no evidence of regression.

In the HK9 colon group the response was complete and the residual tiny nodules were found on histology to consist of

TABLE I.—*Tumour treatment responses*

| | Change in tumour volume percentage (average per mouse) Before treatment/10–15 days after treatment | | | | | |
|---|---|---|---|---|---|---|
| | Colon | | | | | |
| | HK1 | HK6 | HK7 | HK9 | Lung P246 | Breast S32 |
| Controls | 162 (5)* | 150 (7) | 323 (3) | 140 (8) | 150 (9) | 129 (9) |
| Diphtheria toxin (40 $\mu$g/kg) | 50 (6) | 45 (12) | 45 (4) | 19 (16) | 145 (10) | 60 (6) |
| X-rays (100 Gy) | | | | | 84 (6) | 85 (6) |
| CTX (200 mg/kg) | | | | | | 98 (8) |
| HMM (100 mg/kg for 5 days) | | 122 (6) | | | 51 (5) | |

* In parentheses, number of mice.

necrotic debris and granulation tissue only. In other groups there was often evidence of groups of normal-looking cells within large areas of amorphous necrotic debris, and regrowth subsequently often occurred.

Less dramatic tumour responses occurred following irradiation, though on average, size reduction of 15% was usually obtained within 2 weeks.

The effects of HMM and CTX were varied and generally smaller. There was usually a size reduction of 50% in the p246 group but in the S32 and HK6 groups the responses were small and less frequent. In no case was complete remission obtained by these drugs.

FIG. 2.—CEA blood levels in controls: intra-assay error is shown in some cases.

*Effects of CEA levels*

*Controls.*—A random daily variation was seen in individual mice in each tumour group; example Fig. 2. Although this was usually small, in some mice this could sometimes be as high as 60%. The range of variation appeared to be independent of individual CEA blood levels, individual tumour size, and the frequency of venesection.

*Treatment groups.*—The overall response of CEA levels following treatment is summarized in Table II. Examples of individual groups are illustrated in Figs 3–5. It will be readily apparent that the most dramatic responses occurred following diphtheria toxin which reflects the overall tumour response. In many cases peak elevation of plasma CEA levels more than 10-fold occurred, often exceeding the range of sensitivity of the assay. There were few examples of peak elevation, however, in response to cytotoxic drugs, in which the most noticeable effect was a considerable increase in daily variation (often by as much as 100%) and this appeared to be independent of tumour response.

There were several examples in which paradoxical responses of CEA in relation to tumour response occurred. In particular, the most responsive tumour group to diphtheria toxin was the HK9 colon group, which was associated with the least uniform or extensive in terms of peak CEA elevations, but CEA eventually completely disappeared from the blood. This was demonstrated to a lesser degree following radiation to the S32 group, and following cytotoxic drugs in HK6 as well as S32 groups.

There were temporal differences accord-

TABLE II.—*Incidence of peak elevations of CEA*

| | Extent of increase (multiple) | Percentage mice with increased CEA levels | | | | | |
|---|---|---|---|---|---|---|---|
| | | Colon | | | | Lung | Breast |
| | | HK1 | HK6 | HK7 | HK9 | P246 | S32 |
| Diphtheria toxin | >3× | 66 (6)* | 75 (12) | 25 (4) | 19 (16) | 0 (10) | 85 (7) |
| X-ray | >2× | | | | | 50 (6) | 66 (6) |
| CTX | >2× | | | | | | 25 (8) |
| HMM | >2× | 20 (6) | | | | 60 (5) | |

* In parentheses, number mice.

FIG. 3.—CEA blood levels following diphtheria toxin (in parentheses, percentage change in tumour volume).

ing to the treatment used. Following diphtheria toxin, rises in CEA became apparent usually in the first 12–24 h, and seldom within the first 6 h, or after 48 h. In most cases CEA would then remain at these high peaks for 24–48 h, but occasionally would persist for up to 5 days. The subsequent fall in CEA was rapid, usually returning to pretreatment levels in 24 h and thereafter gradually to zero over a further 2–4 days in almost all cases. In contrast, after radiotherapy, there was often a 48 h delay before rises in CEA became apparent, and the subsequent fall in CEA below pretreatment titres tended to be more prolonged, with CEA usually detectable in the blood even at 17 days. In the tumour group responding to cytotoxic drugs (p246) there was a gradual trend of CEA downwards becoming apparent at the 5th day. Unfortunately it was not possible to continue CEA estimations for longer, because the effect of further marrow suppression on already immune-suppressed animals delayed recovery from the anaemia of serial venesection too long to permit further study.

### DISCUSSION

Previous *in vitro* experiments have provided some support for the hypothesis that CEA may be released as a result of tumour lysis. Several workers have shown that CEA is released from cells during the stationary phase of the cell cycle (Drewinko & Yang, 1976, 1980; Goldenberg *et al.*, unpub.). Ellison *et al.* (1977) and

FIG. 4.—CEA blood levels after X-rays (p246 lung) (in parentheses, percentage change in tumour volume).

FIG. 5.—CEA levels after HMM (p246 lung) (in parentheses, percentage change in tumour volume).

Goldenberg *et al.* (unpub.) originally demonstrated that when toxic conditions were introduced to cell lines, up to a 25-fold increase in CEA would appear in culture media over a short interval, whereas when growth rate was accelerated, by increasing the temperature or adding cyclic AMP, a reduction in CEA occurred, and this has been further confirmed more recently by Drewinko & Yang (1980). By implication, CEA release would appear to be maximal when the "health" of the cell was compromised.

The human *in vivo* responses supporting this hypothesis which were described in the introduction should, however, be regarded with some circumspection, because there may be many other processes operating.

The demonstration in this study of numerous instances when effective tumour lysis resulted in peak elevations of circulating CEA, does suggest that tumour necrosis and/or factors associated with this process may play a part in the release of CEA. However, in extrapolating from data of these xenograft studies, it is essential to appreciate that the same may not apply in patients. The conditions were grossly artificial and the methods for inducing tumour necrosis were extreme. As yet no such treatment as effective as diphtheria toxin on the xenograft model is available to clinical practice.

It was surprising, however, that in the HK9 colon tumours, which were by far the best responders to diphtheria toxin, the changes in blood CEA were the least remarkable among the whole colon (HK) tumour group.

The concentration of CEA in the circulation is likely to be maintained in a state of dynamic equilibrium by a set of complex factors affecting the source of CEA in the tissues, the processes by which it is released from tumour tissue into the blood, and its subsequent metabolism. Whilst tumour lysis may be a factor responsible for elevated blood CEA levels, it would seem unlikely to make a major contribution to the many other factors, unless the process were extensive and rapid. The possibility should continue to be entertained, however, that tumour lysis may in part be responsible for the discordant changes which take place in patients during chemo- and radiotherapy.

The author wishes to acknowledge his gratitude and appreciation to the Breast Unit, Royal Marsden Hospital, London SW3, for financial support; to Professors A. J. S. Davies and A. M. Neville for advice and encouragement; and to Dr M. Omerod, Miss N. Neylon, Mr M. Capp and Mr K. Gomer for technical assistance.

### REFERENCES

BORTHWICK, N. M., WILSON, D. W. & BELL, P. A. (1977) Carcinoembryonic antigen (CEA) in patients with breast cancer. *Eur. J. Cancer*, **13**, 171.

CARTER, S. K. (1976) Integration of chemotherapy into combined modality treatment of solid tumours. VII. Adenocarcinoma of the breast. *Cancer Treat. Rev.*, **3**, 141.

DONALDSON, E., VAN NAGELL, J. R., WOOD, E. G., PLETSCH, Q. & GOLDENBERG, D. M. (1976) Carcinoembryonic antigen in patients treated with radiation therapy for invasive squamous cell carcinoma of the uterine cervix. *Am. J. Roentgenol.*, **127**, 829.

DREWINKO, B. & YANG, L. Y. (1976) Restriction of CEA synthesis to the stationary phase of growth of cultured colon carcinoma cells. *Exp. Cell Res.*, **101**, 414.

DREWINKO, B. & YANG, L. Y. (1980) Observations on the synthesis of carcinoembryonic antigen by an established human colonic carcinoma cell line. *Oncology*, **37**, 336.

ELLISON, M. L., LAMB, D., RIVETT, J. & NEVILLE, A. M. (1977) Quantitative aspects of carcinoembryonic antigen output by a human lung carcinoma cell line. *J. Natl Cancer Inst.*, **59**, 309.

KHOO, S. K. & MACKAY, E. V. (1976) Carcinoembryonic antigen (CEA) in ovarian cancer: Factors influencing its incidence and changes which occur in response to cytotoxic drugs. *Br. J. Obstet. Gynaecol.*, **83**, 753.

MAYER, R. J., GARNICK, M. B., STEELE, G. D. & ZAMCHECK, N. (1978) Carcinoembryonic antigen (CEA) as a monitor of chemotherapy in disseminated colorectal cancer. *Cancer* ,**42**, 1428.

MITCHLEY, B. C. V., CLARKE, S., CONNORS, T. A., CARTER, S. M. & NEVILLE, A. M. (1977) Chemotherapy of human tumours in T-lymphocyte-deficient mice. *Cancer Treat. Rep.*, **61**, 451.

NOWAK, K., PECKHAM, M. J. & STEEL, G. G. (1978) Variation in the response of xenografts of colorectal carcinoma to chemotherapy. *Br. J. Cancer*, **37**, 576.

QUAYLE, J. B. (1982) Ability of CEA blood levels to reflect tumour burden: A study in a human graft model. *Br. J. Cancer*, **46**, 220.

RAVRY, M. & MOERTEL, C. G. (1974) Usefulness of serial serum carcinoembryonic antigen (CEA) determinations during anticancer therapy or long term follow-up of gastrointestinal carcinoma. *Cancer*, **34**, 1230.

SHANI, A., O'CONNELL, M. J., MOERTEL, C. G., SCHUTT, A. J., SILVERS, A. & GO, V. L. W. (1978) Serial plasma carcinoembryonic antigen measurements in management of metastatic colorectal carcinoma. *Ann. intern. Med.*, **88**, 627.

SKARIN, A. T., DELWICHE, R., ZAMCHEC, N., LOKICH, J. L. & FREI, E., III (1974) Carcinogembryonic antigen: Clinical correlation with chemotherapy for metastatic gastrointestinal cancer. *Cancer*, **33**, 1239.

STEWARD, A. M., NIXON, D., ZAMCHECK, N. & AISENBERG, A. (1974) Carcinoembryonic antigen in breast cancer patients: Serum levels and disease progress. *Cancer*, **33**, 1246.

SUGARBAKER, P. H., BLOOMER, W. D., CORBETT, E. D. & CHAFFEY, J. J. (1978) Carcinoembryonic antigen (CEA): Its role as a monitor for radiation therapy for colorectal cancer. *Cancer*, **42**, 1434.

WAALKES, T. P., ABELOFF, M. D., WOO, K. B. & ETTINGER, D. S. (1980) Carcinoembryonic antigen for monitoring patients with small cell carcinoma of lung during treatment. *Cancer Res.*, **40**, 4420.

YOUNG, W. L., KASHMIRI, R., HAZEN, Z. R. & MEEKER, W. R., JNR (1976) Usefulness of serial CEA determinations in monitoring chemotherapy. *South. Med. J.*, **69**, 1274.